# Association of PD‐L1 expression with driver gene mutations and clinicopathological characteristics in non‐small cell lung cancer: A real‐world study of 10 441 patients

**DOI:** 10.1111/1759-7714.15244

**Published:** 2024-03-08

**Authors:** Gonzalo Ruiz, Diego Enrico, Yamil D. Mahmoud, Alan Ruiz, María Florencia Cantarella, Laura Leguina, Mariana Barberis, Asunción Beña, Esteban Brest, Solange Starapoli, Andrea Mendoza Bertelli, Florencia Tsou, Carmen Pupareli, María Pía Coppola, Alejandra Scocimarro, Susana Sena, Patricio Levit, Aldo Perfetti, Enrique Aman, María Romina Girotti, Oscar Arrieta, Claudio Martín, Rubén Salanova

**Affiliations:** ^1^ Pathology & Molecular Biology Laboratories Biomakers Buenos Aires Argentina; ^2^ Thoracic Oncology Unit, Department of Medical Oncology Alexander Fleming Cancer Institute Buenos Aires Argentina; ^3^ Clinical Research Unit, Department of Medical Oncology Alexander Fleming Cancer Institute Buenos Aires Argentina; ^4^ Universidad Argentina de la Empresa (UADE), Instituto de Tecnología (INTEC) Buenos Aires Argentina; ^5^ Laboratorio de Glicomedicina, Instituto de Biología y Medicina Experimental (IBYME) Consejo Nacional de Investigaciones Científicas y Técnicas (CONICET) Buenos Aires Argentina; ^6^ Medical Oncology Unit Hospital Zonal Especializado en Agudos y Crónicos Dr. Antonio Cetrangolo Buenos Aires Argentina; ^7^ Medical Oncology Department Hospital Alemán Buenos Aires Argentina; ^8^ Medical Oncology Unit Unión Personal‐Accord Salud Buenos Aires Argentina; ^9^ Medical Oncology Department Centro de Educación Médica e Investigaciones Clínicas (CEMIC) Buenos Aires Argentina; ^10^ Medical Oncology Unit, Swiss Medical Group Buenos Aires Argentina; ^11^ Head of Thoracic Oncology Unit Unidad Funcional de Oncología Torácica, Instituto Nacional de Cancerología (INCan) Mexico City Mexico

**Keywords:** programmed death‐ligand 1 (PD‐L1), non‐small cell lung cancer (NSCLC), immunohistochemistry (IHC), driver mutations, genomic alterations

## Abstract

**Background:**

Programmed death ligand‐1 (PD‐L1) expression is a well‐known predictive biomarker of response to immune checkpoint blockade in non‐small cell lung cancer (NSCLC). However, there is limited evidence of the relationship between PD‐L1 expression, clinicopathological features, and their association with major driver mutations in NSCLC patients in Latin America.

**Methods:**

This retrospective study included patients from Argentina with advanced NSCLC, and centralized evaluation of PD‐L1 expression concurrently with genomic alterations in the driver genes *EGFR*, *ALK*, *ROS1*, *BRAF*, and/or *KRAS* G12C in FFPE tissue samples.

**Results:**

A total of 10 441 patients with advanced NSCLC were analyzed. Adenocarcinoma was the most frequent histological subtype (71.1%). PD‐L1 expression was categorized as PD‐L1 negative (45.1%), PD‐L1 positive low‐expression 1%–49% (32.3%), and PD‐L1 positive high‐expression ≥50% (22.6%). Notably, current smokers and males were more likely to have tumors with PD‐L1 tumor proportion score (TPS) ≥50% and ≥ 80% expression, respectively (*p* < 0.001 and *p* = 0.013). Tumors with non‐adenocarcinoma histology had a significantly higher median PD‐L1 expression (*p* < 0.001). Additionally, PD‐L1 in distant nodes was more likely ≥50% (OR 1.60 [95% CI: 1.14–2.25, *p* < 0.01]). In the multivariate analysis, *EGFR*‐positive tumors were more commonly associated with PD‐L1 low expression (OR 0.62 [95% CI: 0.51–0.75], *p* < 0.01), while ALK‐positive tumors had a significant risk of being PD‐L1 positive (OR 1.81 [95% CI: 1.30–2.52], *p* < 0.01).

**Conclusions:**

PD‐L1 expression was associated with well‐defined clinicopathological and genomic features. These findings provide a comprehensive view of the expression of PD‐L1 in patients with advanced NSCLC in a large Latin American cohort.

## Graphical abstract

There is limited evidence of the relationship between programmed death ligand‐1 (PD‐L1) expression, clinicopathological features, and their association with major driver mutations in non‐small cell lung cancer (NSCLC) patients in Latin America. We evaluated a cohort of 10 441 patients with advanced NSCLC. Current smokers and males were more likely to have tumors with PD‐L1 tumor proportion score (TPS) ≥50% and ≥80% expression. In the multivariate analysis, *EGFR*‐positive tumors were more commonly associated with PD‐L1 low expression, while ALK‐positive tumors had a significant risk of being PD‐L1 positive.

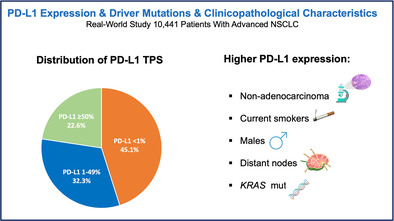

## INTRODUCTION

Treatment with immune checkpoint inhibitors (ICIs) directed against the PD‐1/PD‐L1 axis has revolutionized cancer treatment, especially achieving substantial success in the management of patients with advanced non‐small cell lung cancer (NSCLC).^1^ The expression of PD‐L1, measured by immunohistochemistry as the tumor proportion score (TPS), is defined as the percent of PD‐L1‐positive tumor cells in the tumor tissue. PD‐L1 expression is the primary clinically essential predictive biomarker for anti‐PD‐1/PD‐L1 treatment efficacy in NSCLC. Scientific evidence has shown that high PD‐L1 expression levels are associated with improved survival in patients with advanced NSCLC treated with immunotherapy.^2^, ^3^, ^4^, ^5^ However, PD‐L1 expression is incomplete and imperfect as a stand‐alone biomarker since only a subgroup of patients has long‐term clinical benefit and survival when treated with immune checkpoint inhibitors.^6^

In some tumor models, PD‐L1 expression can be stimulated by tumor extrinsic signals such as interferon‐gamma, or tumor intrinsic signals such as activation of the mammalian target of rapamycin (mTOR), and mitogen‐activated protein kinase (MAPK) signaling pathways.^7^, ^8^, ^9^ However, the main factors associated with its expression at baseline are not fully understood.

It is well known that NSCLC is a heterogeneous disease. Nonsquamous tumors in particular are characterized by subsets of driver genomic alterations capable of being druggable by tyrosine‐kinase inhibitors, including *EGFR*, *KRAS*, *BRAF*, *MET*, and *ERBB2* mutations or *ALK*, *ROS1*, *RET*, and *NTRK* genomic rearrangements.^10^ Critically, the intricate interplay between genomic alterations and PD‐L1 expression in NSCLC is currently under investigation. Recent reports suggest that activating genomic alterations in *KRAS*, *EGFR*, and *ALK*, as well as loss of *PTEN*, possess the potential to biologically influence PD‐L1 expression in NSCLC.^8^, ^11^, ^12^, ^13^ Nevertheless, the association between these factors has only been examined in a limited number of studies, leading to a gap in understanding. Furthermore, inconsistencies have been observed in certain meta‐analyses that have attempted to evaluate this relationship. These variations in findings may be attributed to the heterogeneity among the included studies, which employed different antibodies and utilized varying cutoff levels for defining PD‐L1 expression.^14^, ^15^, ^16^

Interestingly, there is clinical evidence that suggests potential variations in PD‐L1 expression and the effectiveness of immune checkpoint inhibitors based on ethnicity.^17^, ^18^, ^19^ However, validating this hypothesis has proven challenging as most studies investigating the clinicopathological features of NSCLC and PD‐L1 expression have primarily focused on patients from North America, Europe, and Asia, while Latin American and African populations have been notably underrepresented.^20^, ^21^

Given that there is a significant interest in a better understanding of the role of immunotherapy in oncogenic driven‐NSCLC, it becomes crucial to comprehensively assess the factors linked to PD‐L1 expression. This understanding could provide valuable insights into the mechanisms underlying primary response or resistance to immunotherapy. Thus, our study aimed to explore the potential association between PD‐L1 expression and major driver gene alterations, including *EGFR*, *KRAS*, *BRAF*, *ALK*, and *ROS1*, as well as clinicopathological features. This investigation was conducted on a large cohort of patients with advanced NSCLC from a Latin American country.

## METHODS

### Study population

This retrospective study included consecutive patients with advanced NSCLC, and effective evaluation of PD‐L1 expression concurrently with analysis of *EGFR*, *ALK*, *ROS1*, *BRAF*, and/or *KRAS* mutations from available formalin‐fixed paraffin‐embedded (FFPE) samples. Patients were selected from February 2018 to September 2021 from the institutional databases of Biomarkers Inc., which centrally analyzed lung cancer tissue samples from multiple hospitals and cancer centers in Argentina. Data, including basic demographic as well as pathological characteristics, were collected from the same database based on the reports provided by clinicians.

### PD‐L1 assay

PD‐L1 immunohistochemistry (IHC) testing was performed using the PD‐L1 clone 22C3, and the pharmDx kit, and Dako Automated Link 48 platform (Dako). PD‐L1 expression measured as TPS was calculated as the percentage of positive cells in at least 100 viable tumor cells with complete or partial membrane staining assessed by four experienced pathologists (LL, MB, MAB, and GGR).^22^ In instances of discrepancy, a consensus meeting involving a fifth senior pathologist was convened to reach an agreement, and the kappa coefficient was used. A PD‐L1 TPS <1% was defined as negative, and a PD‐L1 TPS ≥1% was considered positive. Additionally, PD‐L1 positive samples were stratified as low PD‐L1 expression (PD‐L1 TPS 1%–49%) and high PD‐L1 expression (PD‐L1 TPS ≥50%).

### Driver mutation analyses

The assessment of *EGFR*, *BRAF*, and *KRAS* p.G12C genomic alterations was performed by extracting genomic DNA from FFPE tumor tissue using a QIAMP mini DNA kit (Qiagen) at the QIAcube instrument (Qiagen), according to the manufacturer's instructions. *EGFR* mutations were detected using the AmoyDX *EGFR* 29 mutations detection kit (AmoyDx) at the Rotor‐Gene Q instrument (Qiagen), and *EGFR* mutation analysis kit (Entrogen) at Cobas z480 instrument (Roche).^23^, ^24^ Both kits are designed for real‐time PCR assays for the qualitative detection of mutations of the *EGFR* gene (LRG_304t1) (Supplementary Methods). *BRAF* mutations were assessed using *BRAF* codon 600 mutation analysis real‐time PCR kit (Entrogen) of exon 15 (Supplementary Methods).^25^ *KRAS* p.G12C mutation was tested by AmoyDX *KRAS* mutation detection kit, real‐time PCR Kit (AmoyDx) at the Cobas z480 instrument (Roche).^26^ *ALK* fusion testing was performed with a fully automated IHC assay using a D5F3 clone (Ventana Roche). D5F3 was additionally assessed using the OptiView enhanced detection and amplification system. ALK‐positive cases were interpreted using Ventana ALK (D5F3) CDx Assay (Roche).^27^ ROS1 fusion testing was performed in *ALK*‐negative cases. ROS1 fusion was analyzed by IHC (D4D6 clone, Cell Signaling Technology) and confirmed by FISH (ZytoLight SPEC ROS1 Dual Color Break Apart Probe) (Supplementary Methods).^28^, ^29^

### Statistical analysis

Categorical variables are summarized using frequency and percentage, while continuous variables are described by their median, standard deviation, or interquartile range (IQR). Associations with qualitative variables were assessed using the Chi‐square or Fisher's exact test, and for quantitative variables, analysis of variance (ANOVA) or the Kruskal‐Wallis test was employed. Pairwise comparisons between groups were conducted using the Wilcoxon test, and *p*‐values were adjusted using the Holm method. The multivariate analysis of PD‐L1 expression utilized a logistic regression model, and the results are reported as adjusted odds ratios (OR). All statistical analyses were performed using R software (version 4.3.0). Two‐tailed tests and *p*‐values <0.05 were used to determine statistical significance.

## RESULTS

### Patient clinicopathological characteristics

**FIGURE 1:**
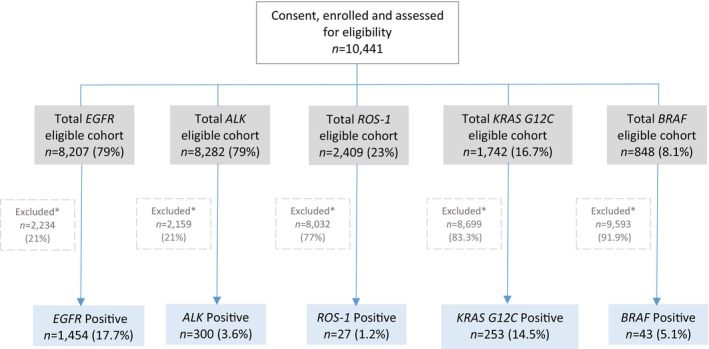
Flow chart of included patients and biomarker analysis. *Patients excluded represented samples nontested or not evaluable.

A total of 10 441 patients with advanced NSCLC were included for analysis (Figure 1). Among the total population, PD‐1 was successfully evaluated in 8977 (86%) cases. Patient characteristics are summarized in Table 1. The majority of patients were male (5176 patients, 58%), and the median age at diagnosis was 66 years (SD 10.5). Lung adenocarcinoma was the most frequent histological type (6388, 71.1%). Among patients with available data, distribution according to smoking status were for never smokers, former and current smokers 325 (21.6%) 541 (35.9%), a 641 (42.5%), respectively. Of note, this cohort included predominantly biopsies from thoracic sites (lung primary tumor [5764 patients, 64.2%], and metastases [1675 patients, 18.7%]).

**TABLE 1:** Patient characteristics and driver alterations according to PD‐L1 expression.

Characteristics no, (%)	Overall	PD‐L1 negative	PD‐L1 1–49%	PD‐L1 ≥ 50%	*p*‐value
	*N* = 8977	*N* = 4051	*N* = 2895	*N* = 2031	
Sex					0.21
Female	3801 (42%)	1700 (42%)	1263 (44%)	838 (41%)	
Male	5176 (58%)	2351 (58%)	1632 (56%)	1193 (59%)	
Age					0.14
Mean (SD)‐year	66 (10.5)	66 (10.7)	66 (10.4)	65 (10.1)	
Histological types					<0.001
Adenocarcinoma	6388 (71.1%)	3051 (75.3%)	2032 (70.1%)	1305 (64.2%)	
Squamous	1173 (13.2%)	444 (11%)	429 (14.8%)	300 (15%)	
Large cell	8 (0.09%)	2 (0.05%)	3 (0.1%)	3 (0.1%)	
Adenosquamous	29 (0.33%)	11 (0.3%)	9 (0.3%)	9 (0.4%)	
NSCLC NOS	1287 (14.3%)	493 (12.1%)	405 (14%)	389 (19.1%)	
Not available	92 (1.02%)	50 (0.55%)	17 (0.19%)	25 (0.27%)	
Biopsy site					<0.001
Primary tumor	5764 (64.2%)	2693 (66.5%)	1831 (63.2%)	1240 (61.1%)	
Metastasis	1675 (18.7%)	780 (19.3%)	512 (17.7%)	383 (18.9%)	
Regional nodes	1180 (13.1%)	457 (11.3%)	438 (15.1%)	285 (14.0%)	
Distant nodes	358 (4.0%)	121 (3.0%)	114 (3.9%)	123 (6.1%)	
Smoking status^a^					<0.001
Nonsmoker	325 (21.6%)	140 (22.2%)	131 (25.0%)	54 (15.3%)	
Former smoker	541 (35.9%)	210 (33.3%)	201 (38.4%)	130 (36.8%)	
Current smoker	641 (42.5%)	280 (44.4%)	192 (36.6%)	169 (47.9%)	
Not available	7470 (83,2%)	3421 (84.5%)	2371 (81.9%)	1678 (82.6%)	
*EGFR* ^a^					<0.001
Negative	5800 (82.5%)	2559 (81.0%)	1847 (80.7%)	1394 (88.0%)	
Positive	1232 (17.5%)	599 (19.0%)	443 (19.3%)	190 (12.0%)	
Not tested	1945 (21.7%)	893 (22.0%)	605 (20.9%)	447 (22.0%)	
*ALK* ^a^					<0.001
Negative	7001 (96.3%)	3246 (97.3%)	2218 (95.3%)	1537 (95.5%)	
Positive	270 (3.7%)	89 (2.7%)	109 (4.7%)	72 (4.5%)	
Not tested	1706 (19%)	716 (17.7%)	568 (19.6%)	433 (20.8%)	
*BRAF* ^a^					0.24
Negative	749 (95.1%)	410 (96.2%)	320 (93.6%)	19 (95.0%)	
Positive	39 (4.9%)	16 (3.8%)	22 (6.4%)	1 (5.0%)	
Not tested	8189 (91.2%)	3625 (89.5%)	2553 (88.2%)	2011 (99.0%)	
*ROS1* ^a^					0.052
Negative	2084 (98.7%)	944 (99.4%)	641 (98.3%)	499 (98.0%)	
Positive	27 (1.3%)	6 (0.6%)	11 (1.7%)	10 (2.0%)	
Not tested	6866 (76,5%)	3101 (76.6%)	2243 (77.5%)	1522 (74.9%)	
*KRAS*_G12C^a^					0.003
Negative	1376 (85.5%)	584 (86.5%)	494 (87.7%)	298 (80.1%)	
Positive	234 (14.5%)	91 (13.5%)	69 (12.3%)	74 (19.9%)	
Not tested	7367 (82,1%)	3376 (83.3%)	2332 (80,6%)	1659 (81.7%)	

*Note*: *χ* ^2^‐test, One‐way ANOVA, and Fisher's exact test. *p*‐value was calculated for patients with available data.

Abbreviations: ANOVA, analysis of variance; NSCLC NOS, non‐small cell lung not otherwise specified; PD‐L1, programmed death ligand‐1; SD, standard deviation.

^a^ Percentages were calculated considering the available data and molecular test performed. Nonsmoker was defined as those who have never smoked, or who have smoked less than 100 cigarettes in their lifetime.

### PD‐L1 expression and clinical features

**FIGURE 2:**
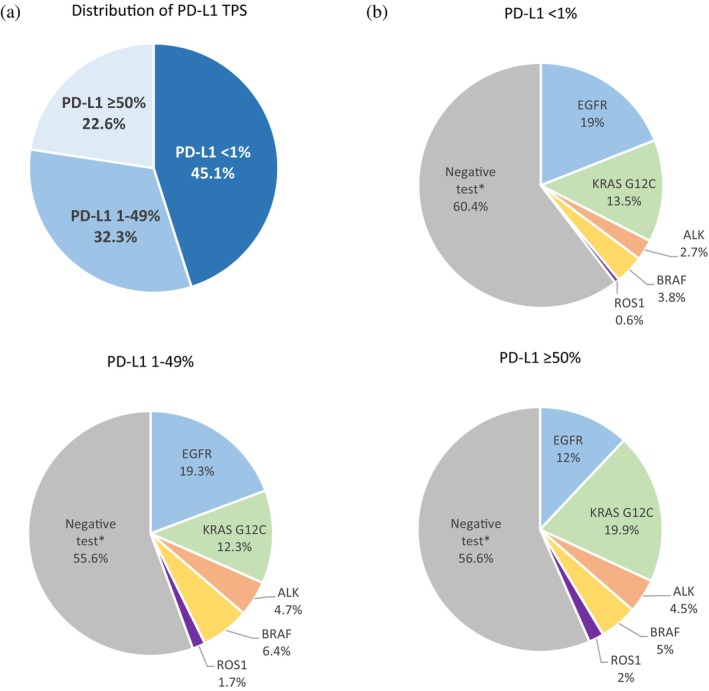
Frequency of programmed death ligand‐1 (PD‐L1) expression in the total population (a). Frequency of driver oncogene alterations (*EGFR*, *KRAS G12C*, *BRAF*, *ALK*, and *ROS1*) according to the expression of PD‐L1 (b). *Negative tests represent tumors without *EGFR*, *KRAS G12C*, *BRAF*, *ALK*, and *ROS1* alterations. Only tumors with available test analysis were included in these graphs.

**FIGURE 3:**
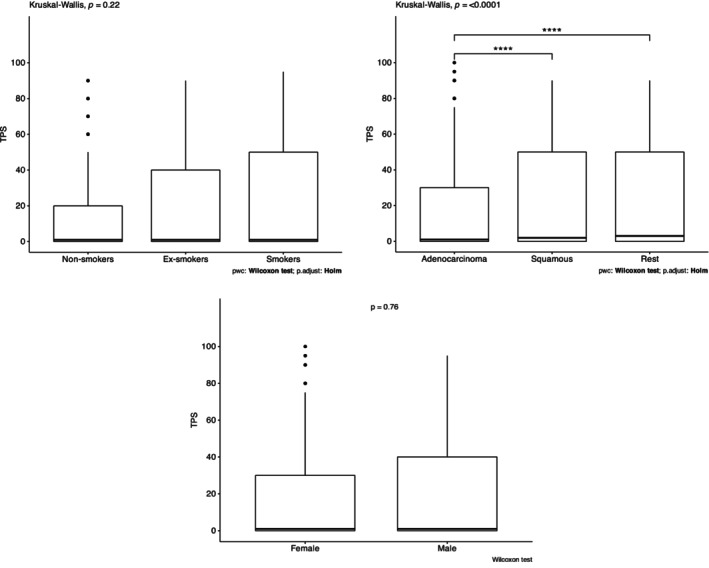
Association of programmed death ligand‐1 (PD‐L1) expression in tumor cells (TPS) with clinicopathological characteristics. Statistical significance: ****p < 0.0001.

PD‐L1 expression was categorized as PD‐L1 negative (4051, 45.1%), low‐expression 1%–49% (2895, 32.3%), and high‐expression ≥50% (2031, 22.6%) (Figure 2a). Considering the subgroup of tumors with high PD‐L1 expression, those with PD‐L1 TPS 50–80 (15.2%) were more common than >80 (7.3%) (Table S1). Although no difference was observed in median PD‐L1 expression according to smoking status and gender, current smokers and male patients more likely had tumors with PD‐L1 TPS ≥50% and ≥ 80% expression, respectively (*p* < 0.001 and *p* = 0.013). Tumors with non‐adenocarcinoma histology had a significantly higher median PD‐L1 expression (*p* < 0.0001) (Figure 3). Additionally, the multivariate analysis showed that samples taken from metastatic lesions had a significantly lower risk of being PD‐L1 positive and PD‐L1 ≥ 50% expression (OR 0.71 [95% CI: 0.52–0.79], *p* < 0.01, and OR 0.79 [95% CI: 0.59–0.99], *p* = 0.03, respectively) (Table 2). Contrary, the score of PD‐L1 TPS in distant nodes was more likely ≥50% (OR 1.60 [95% CI: 1.14–2.25, *p* < 0.01]).

**TABLE 2:** Impact of driver alterations status on the PD‐L1 expression.

	PD‐L1 + (*N* = 4864)		PD‐L1 ≥ 50% (*N* = 4864)	
	OR, 95% CI	*p*‐value	OR, 95% CI	*p*‐value
Age	1.00 (1.00–1.01)	0.20	1.00 (0.99–1.00)	0.37
Histological types				
Adenocarcinoma	‐		‐	
Adenosquamous	1.24 (0.47–3.28)	0.70	1.46 (0.51–4.17)	0.48
Squamous	1.38 (0.94–2.00)	0.10	1.17 (0.76–1.80)	0.46
Large cell	2.33 (0.45–12.1)	0.30	2.67 (0.59–12.0)	0.20
NSCLC NOS	1.43 (1.21–1.70)	<0.01	1.49 (1.24–1.79)	<0.01
Biopsy site				
Regional nodes	‐		‐	
Primary tumor	0.67 (0.57–0.80)	<0.01	0.83 (0.68–1.01)	0.06
Metastasis	0.71 (0.52–0.79)	<0.01	0.79 (0.59–0.99)	0.03
Distant nodes	1.22 (0.87–1.70)	0.20	1.60 (1.14–2.25)	<0.01
*EGFR*				
Negative	‐		‐	
Positive	0.91 (0.78–1.06)	0.20	0.62 (0.51–0.75)	<0.01
*ALK*				
Negative	‐		‐	
Positive	1.81 (1.30–2.52)	<0.01	1.11 (0.78–1.57)	0.57

*Note*: Multivariate logistic regression. Multivariate logistic regression model; data presented by adjusted odds ratio. *KRAS*, *BRAF*, and *ROS1* analyses were excluded given the low number of cases with complete data.

Abbreviations: CI, confidence interval; NOS, not otherwise specified; OR, odds ratio; PD‐L1, programmed death ligand 1.

### Association between driver gene mutations and PD‐L1 expression

**FIGURE 4:**
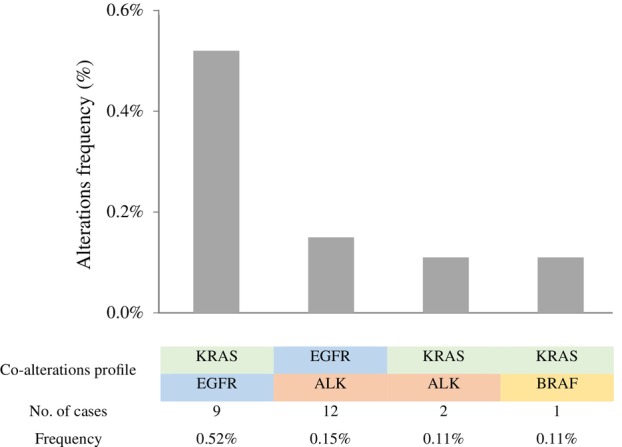
Frequency of cases harboring concomitant alteration.

Among patients with evaluable tests for the five molecular alterations tested, *EGFR* mutation was the most common alteration (17.7%), followed by *KRAS* p.G12C (14.5%), *BRAF* mutation (5.1% [p.V600E 3.6%]), *ALK* fusion (3.6%), and *ROS1* fusion (1.2%) (Figure 1). The frequency of concomitant alteration according to the tested cases was *EGFR* + *ALK* in 12 patients (0.15%), *KRAS* p.G12C + *EGFR* in nine (0.52%) patients, *KRAS* p.G12C + ALK in two (0.11%), and *KRAS* p.G12C + *BRAF* p.V600E in one (0.11%) case (Figure 4). Remarkably, a total of 135 (9.3%) non‐adenocarcinoma tumors harbored *EGFR* mutations, including 10 with squamous cell carcinoma, two with mixed histology, and 123 with carcinoma not otherwise specified, that were tested given the clinical indication.

The distribution of PD‐L1 expression and driver mutation status is summarized in Figure 2b. In the multivariate logistic regression analysis, *EGFR* mutated tumors were more commonly PD‐L1 low (OR 0.62 [95% CI: 0.51–0.75], *p* < 0.01), and the median TPS was 1% (IQR 0–15) (Table 2). On the contrary, *ALK* translocated tumors had a significant risk of being PD‐L1 positive (OR 1.81 [95% CI: 1.30–2.52], *p* < 0.01), and the median TPS for *ALK* tumors was 5% (IQR 0–50).

Tumors with *KRAS*, *BRAF*, and *ROS1* genomic alterations were excluded from the multivariate analysis due to the limited number of cases with available data. However, a univariate association between PD‐L1 expression and *KRAS* status was found since *KRAS*‐mutated tumors were more likely PD‐L1‐high than PD‐L1 low (19.9% vs. 12.3%, *p* = 0.003). Contrarily, no statistical associations were found between *ROS1* or *BRAF* and PD‐L1 status (*p* = 0.052 and *p* = 0.240, respectively).

## DISCUSSION

The administration of immune checkpoint inhibitors targeting anti‐PD‐1 or PD‐L1 has demonstrated enhanced survival outcomes in individuals with metastatic NSCLC. However, it is important to note that treatment benefits have only been observed in a specific subgroup of patients, and the identification of predictive factors for immunotherapy response remains under intensive research.^4^ Although imperfect, PD‐L1 expression is the only biomarker currently utilized in clinical practice to guide treatment decisions regarding immunotherapy in advanced NSCLC. Nonetheless, the expression of PD‐L1 in NSCLC exhibits considerable variability, and a comprehensive understanding of the factors influencing its expression is yet to be achieved.

Numerous studies have investigated the association between PD‐L1 expression and clinicopathological features in NSCLC. Several meta‐analyses, involving substantial patient cohorts ranging from 7541 to 11 444 individuals, have encountered methodological limitations such as the inclusion of heterogeneous NSCLC populations (excluding patients from Africa and Latin America), and the utilization of various antibodies, staining techniques, and threshold values for defining PD‐L1 expression.^14^, ^15^, ^16^ To the best of our knowledge, our study is unique and represents the largest single‐region real‐world cohort of a centralized PD‐L1 analysis in advanced NSCLC.

In our study, the distribution of PD‐L1 expression in NSCLC was aligned with previous findings of PD‐L1 positivity ranging from 20% to 70% utilizing the same antibody clone.^30^, ^31^, ^32^, ^33^ Remarkably, the predominant subgroup observed was the PD‐L1 negative category. In phase III trials investigating untreated advanced NSCLCs, the prevalence of PD‐L1 TPS negative expression was reported to be between 30.8% and 39.5% using various PD‐L1 antibodies.^34^, ^35^, ^36^, ^37^ Notably, in line with our findings, although not entirely validated, certain studies have reported a positive correlation between elevated PD‐L1 expression and non‐adenocarcinoma histology.^38^, ^39^

Based on our findings, patients who were current smokers exhibited a higher likelihood of having elevated PD‐L1 expression. Consistent with this observation, previous studies have reported similar results, corroborating our findings.^40^ Notably, tobacco smoking is commonly linked to T cell exhaustion and the upregulation of PD‐1, which ultimately contributes to immune evasion.^41^, ^42^ Lung cancer cases in smokers have been noted to exhibit a substantial load of neoantigens, heightened immunogenicity, and upregulation of PD‐L1.^43^ This data is of great significance as several studies have consistently demonstrated that advanced NSCLC patients who are current smokers and exhibit positive PD‐L1 expression are more inclined to respond favorably to anti‐PD‐1 monotherapy in comparison to individuals who have never smoked.^44^, ^45^, ^46^, ^47^

In our study, a remarkably high PD‐L1 expression (≥80%) was notably more prevalent among males than females, potentially attributable to the higher incidence of cigarette smoking in males. While this explanation appears to be the most plausible, the relationship between sex and PD‐L1 expression remains inadequately elucidated. Notably, a meta‐analysis conducted by Zhang et al. revealed that PD‐L1 expression was elevated in males, pooling the results from 11 444 patients.^16^ Opposite, no significant correlation between positive PD‐L1 expression and gender was found in a pooled analysis including 3128 cases performed by Yang et el.^15^ Conversely, the correlation between PD‐L1 expression and gender could potentially be influenced by sex hormones, as emerging evidence suggests that these hormones have the capacity to regulate numerous immune‐related genes, including those involved in the PD‐1/PD‐L1 pathway.^48^, ^49^ The validation of the association between elevated PD‐L1 expression and male patients holds significant clinical relevance, as multiple phase III studies investigating first‐line immune checkpoint inhibitors in advanced NSCLC have demonstrated that anti‐PD‐1/anti‐PD‐L1 monotherapy exhibits greater efficacy in men compared to women.^50^

Nonhomogenous PD‐L1 expression between primary tumor and metastatic sites has previously been reported.^51^, ^52^ The multivariate analysis of our study showed that tissue samples from distant nodes were more likely to have high PD‐L1 expression than those from the primary tumor and regional lymph nodes. Given it was not tested in paired primary and metastatic samples, it is not possible to have strong methodological conclusions. However, it reinforces that PD‐L1 expression in lung cancer could be heterogeneous and dynamic, hence the reliability and feasibility of the PD‐L1 expression on a single biopsy specimen, as a reference for immuno‐oncology treatment, remains controversial.^53^, ^54^

In this new era of genomic characterization of NSCLC, a deeper understanding of the molecular factors associated with PD‐L1 expression can help elucidate mechanisms of primary response and resistance to immunotherapy. In this context, evidence has characterized that *EGFR*‐mutated tumors have a lower tumor mutation burden (TMB), but the association with PD‐L1 expression remains unclear.^38^, ^55^, ^56^, ^57^, ^58^, ^59^ Evans et al. analyzed the PD‐L1 expression among 10 005 patients with NSCLC in the UK and found that classical *EGFR* mutations were associated with lower rates of PD‐L1 expression, and nonclassical *EGFR* mutations were associated with higher rates.^60^ Contrarily, a meta‐analysis conducted by Zhang et al., including 47 studies and 11 444 patients, showed that high PD‐L1 expression was associated with *EGFR* mutations.^16^ In another meta‐analysis performed by Li et al., analyzing 50 studies and 11 383 patients, the pooled results revealed that PD‐L1 expression was related to *EGFR* wild‐type tumors.^40^ Taking advantage of our large homogenous cohort, the multivariate analysis demonstrated that *EGFR*‐mutated tumors more likely had low PD‐L1 expression with a very low median TPS.

Likewise, preclinical studies have demonstrated that *ALK* translocation and its downstream signaling pathways can drive PD‐L1 expression.^13^, ^61^ However, the association between positive PD‐L1 expression and *ALK* status has not been yet validated in clinical studies with contradictory findings.^16^, ^40^, ^62^, ^63^, ^64^ Our results suggest that ALK‐positive tumors have a significantly higher risk of PD‐L1 positive expression.

Our study revealed that *KRAS* p.G12C‐mutant tumors were more likely to have a high PD‐L1 expression in the univariate analysis. Studies on cell lines revealed that *KRAS*‐mutated NSCLC can be regulated by MAPK and partially by STAT3 signaling pathways.^9^, ^65^, ^66^ As a consequence, similar to our findings, several studies and meta‐analyses confirmed the positive correlation between PD‐L1 expression and *KRAS* mutation in NSCLC.^38^, ^40^, ^67^, ^68^

Finally, the analysis of uncommon driver mutations is usually limited by patient numbers. A small number of studies have revealed that *BRAF* mutation, particularly p.V600E, is associated with a high level of PD‐L1 expression.^68^, ^69^ Concerning *ROS1* fusion, no association was found in our study in line with previous reports.^59^, ^70^

Our results should be analyzed with caution considering study limitations. First, the retrospective nature of our analysis resulted in incomplete data for some patients. Second, PD‐1 was unsuccessfully evaluated in 14% of cases as a result of poor tissue quantity and quality. The unsuccessful evaluation of PD‐L1 expression was estimated at around 10% in the real‐world setting and 5% in clinical trials.^34^, ^37^, ^71^, ^72^ Third, analysis of uncommon driver alterations was usually limited by the low number of patients. In our cohort, not all cases were tested for the entire mutational profile (*EGFR*, *KRAS*, *ALK*, *ROS1*, and *BRAF*) which might have affected the multivariate analysis. The main reason for this discrepancy was the heterogeneous biomarker testing reimbursement for each case. Fourth, given that PD‐L1 expression in NSCLC could be heterogeneous and dynamic, the association between a potentially changing variable (PD‐L1 expression), with a constant variable (the mutational profile), might limit the reliability, and reproducibility of the results. However, our study had an advantage over other studies since all the samples were processed and read in the same institution with a consistent antibody, technique, and experienced pathologists.

In conclusion, this is the largest and most homogeneous study analyzing PD‐L1 expression and its association with clinicopathological and genomic alterations in a Latin American cohort. In summary, we found that males and current smokers, as well as tumors with non‐adenocarcinoma histology, *KRAS* mutations, and tissue samples from distant nodes, were associated with high PD‐L1 expression. In contrast, tumors with *EGFR* mutations were more likely to have low PD‐L1 expression. This study, together with the current evidence, is ultimately intended to understand the potential associations between PD‐L1 expression with clinicopathological relevance and genomic alterations.

## AUTHOR CONTRIBUTIONS

Conception and design: Gonzalo Ruiz, Diego Enrico, Andrea Mendoza Bertelli, María Romina Girotti, Yamil D. Mahmoud, Rubén Salanova. Financial support: Rubén Salanova. Administrative support: Rubén Salanova, Claudio Martín. Provision of study materials: Gonzalo Ruiz, Diego Enrico, Yamil D. Mahmoud, Andrea Mendoza Bertelli, María Romina Girotti, Rubén Salanova. Collection and assembly of data: Yamil D. Mahmoud, Gonzalo Ruiz, Diego Enrico, Andrea Mendoza Bertelli, María Romina Girotti, Rubén Salanova. Data analysis and interpretation: Yamil D. Mahmoud, Gonzalo Ruiz, Diego Enrico, Andrea Mendoza Bertelli, Rubén Salanova. Manuscript writing: Diego Enrico, Gonzalo Ruiz, Andrea Mendoza Bertelli, María Romina Girotti. Final approval of manuscript: All authors. Accountable for all aspects of the work: All authors.

## CONFLICT OF INTEREST STATEMENT

The authors declare no conflicts of interest related to this study.

## Supporting information


**Data S1.** Supporting Information.
