# Supplementary material for: Association of PD‐L1 expression with driver gene mutations and clinicopathological characteristics in non‐small cell lung cancer: A real‐world study of 10 441 patients
Source: Thorac Cancer. 2024 Mar 8;15(11):895–905. doi: 10.1111/1759-7714.15244 (PMC11016406; doi:10.1111/1759-7714.15244)
Supplement: Supplementary file 1 — Data S1. Supporting Information. [file TCA-15-895-s001.docx]

Supplementals

**MATERIALS AND METHODS**

1. Driver mutation analyses

***EGFR***

In the AmoyDX EGFR 29 Mutations Detection Kit (AmoyDx), the limit of detection for the variants is: 1% for exon 19 deletions, c.2582T>A p.(Leu861Gln), exon 20 insertions, c.2156G>C p.(Gly719Ala), 2155G>T p.(Gly719Cys), c.2303G>T p.(Ser768Ile), c.2582T>A p.(Leu861Gln) and 2,5% for c.2369C>T p.(Thr790Met) and 2155G>T, p.(Gly719Ser) (1-4).

Entrogen kit detects the following variants: c.2369C>T p.(Thr790Met), exon 19 deletions (48 variants), c.2573T>G, p.(Leu858Arg), c.2582T>A p.(Leu861Gln), codon 719 mutations (4 variants), c.2303G>, p.(Ser768Ile), and exon 20 insertions (3 variant s). The limits of detection for these variants are: 1% for c.2369C>T p.(Thr790Met), exon 19 deletions (48 variants), c.2573T>G, p.(Leu858Arg), c.2582T>A p.(Leu861Gln), 1,5% for c.2303G>, p.(Ser768Ile), 2% for exon 20 insertions and 3,2% for codon 719 variants (5-6).

***BRAF***

*BRAF* mutations were detected using *BRAF* codón 600 Mutation Analysis Kit (Entrogen). This kit it is also a real time assay that detects somatic mutations in exón 15 of BRAF gene. It allows to detect the following variants: c.1799T>A or c.1799_1800delTGinsAA, p.(Val600Glu), c.1798_1799GT>AA, p.(Val600Lys), c.1798_1799GT>AG, p.(Val600Arg), c.1799_1800TG>AT, p.(Val600Asp), c.1798G>A, p.(Val600Met) and c.1799T>G, p.(Val600Gly). This last mutation can not be distinguish from c.1798_1799GT>AG, p.(Val600Arg). The limits of detection for these variants are: 1% for c.1799T>A or c.1799_1800delTGinsAA, p.(Val600Glu), c.1798_1799GT>AA, p.(Val600Lys), c.1798_1799GT>AG, p.(Val600Arg), c.1799_1800TG>AT, p.(Val600Asp) and c.1799T>G, p.(Val600Gly) and 3% for c.1798G>A, p.(Val600Met). These protocols were performed according to manufacturer instructions (7-12).

***ROS1***

*ROS1* testing was performed in cases of *ALK*-negative lung adenocarcinoma. In a first step IHC was done, and in positive cases FISH was used to confirm gene rearrangements. *ROS1* IHC interpretation was evaluated according to “IASLC ATLAS of *ALK-ROS1*” and “ROS1 IHC implementation guide. Roche Diagnostics Belgium 1 Version 1 – June 2020 – MED2020_009” using D4D6 clone (Cell Signaling Technology CST, Danvers, MA).

A practical cutoff value of 15% has been reported in most studies on clinical specimens, and this value has correlated well with the *ROS1* fusion status as determined by RT-PCR. Thus, when the rearrangement-positive cell rate is 15% or more, the specimen is interpreted as being *ROS1*-positive; when the rate is less than 15%, the specimen is interpreted as being *ROS1*-negative.

ZytoLight ® SPEC *ROS1* Dual Color Break Apart Probe was used to perform *ROS1* FISH, according to the datasheet provided. For scoring, 50 tumor cells were evaluated by a pathologist. If 10-30% of the cells had rearrangement, a second pathologist was needed. Positive cases were the ones in which 30% or more tumoral cells showed rearrangements, seen by one pathologist, or 15% or more when seen by two of them (13-15).

***ALK***

Our cases were evaluated for *ALK* according to “VENTANA *ALK* (D5F3) CDx Assay Interpretation Guide for Non-Small Cell Lung Carcinoma (NSCLC)” wich stablishes positive for *ALK* presence of strong granular cytoplasmic staining in tumor cells (any percentage of positive tumor cells) and negative for *ALK* the Absence of strong granular cytoplasmic staining in tumor cells (16-26).

***KRAS***

For screening of *KRAS* G12C mutations we used AmoyDx@ *KRAS* Mutation Detection Kit wich is a real-time PCR assay for qualitative detection of 19 somatic mutations in codons 12, 1 3, 59, 61, 117 and 146 of *KRAS* gene in human genomic DNA extracted from formalin-fixed paraffin-embedded (FFPE) tumor tissue (27-30).

| **Characteristics no, (%)** | **PD-L1 <50%**  **N = 2,895** | **PD-L1 50-80%**  **N = 1,372** | **PD-L1 >80%,**  **N = 659** | **P-value^1^** |
| --- | --- | --- | --- | --- |
| **Sex** |  |  |  | 0.013 |
| Female | 1,263 (44%) | 595 (43%) | 243 (37%) |  |
| Male | 1,632 (56%) | 777 (57%) | 416 (63%) |  |
| **Age** |  |  |  | 0.15 |
| Mean (SD) -yr | 66 (10.4) | 65 (10.3) | 66 (9.8) |  |
| **Histological types** |  |  |  | <0.001 |
| Adenocarcinoma | 2,032 (70.2%) | 901 (65.7%) | 404 (61.3%) |  |
| Squamous | 429 (14.8%) | 186 (13.6%) | 114 (17.3%) |  |
| Large cell | 3 (0.1%) | 2 (0.1%) | 1 (0.2%) |  |
| Adenosquamous | 9 (0.3%) | 6 (0.4%) | 3 (0.5%) |  |
| NSCLC NOS | 405 (14%) | 265 (19.3%) | 124 (18.8%) |  |
| Not available | 17 (0.6%) | 12 (0.9%) | 13 (2%) |  |
| **Biopsy Site** |  |  |  | <0.001 |
| Primary tumor | 1,831 (63.2%) | 838 (61.1%) | 402 (61%) |  |
| Metastasis | 512 (17.7%) | 255 (18.6%) | 128 (19.4%) |  |
| Regional nodes | 438 (15.1%) | 204 (14.9%) | 81 (12.3%) |  |
| Distant nodes | 114 (3.9%) | 75 (5.5%) | 48 (7.3%) |  |
| **Smoking status^#^** |  |  |  | 0.003 |
| Non-smoker | 131 (25%) | 39 (16.5%) | 15 (12.8%) |  |
| Former smoker | 201 (38.4%) | 88 (37.3%) | 42 (35.9%) |  |
| Current smoker | 192 (36.6%) | 109 (46.2%) | 60 (51.3%) |  |
| Not available | 2,371 (82%) | 1,136 (83%) | 542 (82.3%) |  |
| ***EGFR*^#^** |  |  |  | <0.001 |
| Negative | 1,847 (80.7%) | 931 (86.5%) | 463 (91.1%) |  |
| Positive | 443 (19.3%) | 145 (13.5%) | 45 (8.9%) |  |
| Not Tested | 605 (20.9%) | 296 (21.6%) | 151 (22.9%) |  |
| ***ALK*^#^** |  |  |  | <0.001 |
| Negative | 2,218 (95.3%) | 1,045 (95.4%) | 492 (96%) |  |
| Positive | 109 (4.7%) | 51 (4.7%) | 21 (4%) |  |
| Not Tested | 568 (19.6%) | 276 (20.1%) | 146 (22.2%) |  |
| ***BRAF*^#^** |  |  |  | 0.30 |
| Negative | 320 (93.6%) | 15 (93.8%) | 4 (100%) |  |
| Positive | 22 (6.4%) | 1 (6.3%) | 0 (0%) |  |
| Not Tested | 2,553(88.2%) | 1,356 (99%) | 655 (99.4%) |  |
| ***ROS1*^#^** |  |  |  | 0.03 |
| Negative | 641 (98.3%) | 341 (97.4%) | 158 (99.4%) |  |
| Positive | 11 (1.7%) | 9 (2.6%) | 1 (0.6%) |  |
| Not Tested | 2,243 (77.5%) | 1,022 (74.5%) | 500 (75.9%) |  |
| ***KRAS* G12C^#^** |  |  |  | 0.009 |
| Negative | 494 (87.7%) | 200 (80.3%) | 98 (79.7%) |  |
| Positive | 69 (12.3%) | 49 (19.7%) | 25 (20.3%) |  |
| Not Tested | 2,332 (80,6%) | 1,123 (81.9%) | 536 (81.3%) |  |
| Abbreviations: NSCLC NOS, non-small-cell lung not otherwise specified; SD, standard deviation.  Chi-squared test, One-way ANOVA, and Fisher's exact test. P value was calculated for patients with available data.  #Percentages were calculated considering the available data and molecular test performed. Non-smoker was defined as those who has never smoked, or who has smoked less than 100 cigarettes in the lifetime.  ^1^ Pearson's Chi-squared test; One-way ANOVA; Fisher's Exact Test for Count Data with simulated p-value (based on 2000 replicates). | | | | |

**REFERENCES**

1. Shama SV, Bell DW, Settleman J, et al; Epidermal growth factor receptor mutations in lung cancer. Nat Rev Cancer, 2007,7(3): 169-81.
2. Ressel R, Moran T, Queralt C, et al; Screening for epidermal growth factor receptor mutations in lung cancer. N Engl J Med, 2009,361 (10):958-67.
3. Mork Ts, Wu YL, Thongprasert S, et al; Gefitinib or carboplatin-paclitaxel in pulmonary adenocarcinoma. N Engl J Med, 2009,361
4. Gazdar AF; Personalized medicine and inhibition of EGFR signaling in lung cancer. N Engl J Med, 2009, 361 (10): 1018-20.

Benvenuti S. et al., Oncogenic activation of the RAS/RAF signaling pathway impairs the response of metastatic colorectal cancers to anti-epidermal growth factor receptor antibody therapies. Cancer Res. 2007 Mar 15;67(6):2643-8. 2.

Salomon DS. et al., Epidermal growth factor-related peptides and their receptors in human malignancies. Critical Reviews in Oncology/Haematology 1995; 19:183-232

McCubrey JA, Steelman LS, Chappell WH, et al. Roles of the Raf/MEK/ERK pathway in cell growth, malignant transformation and drug resistance. BiochimBiophysActa 2007;1773:1263–1284.

Davies H, Bignell GR, Cox C, et al. Mutations of the BRAF gene in human cancer. Nature 2002; 417:949-54.

Flaherty K.T., et al. Inhibition of Mutated, Activated BRAF in Metastatic Melanoma. The New England Journal of Medicine, 2010; vol.363 no. 9: 809-819.

Joseph E.W., et al., The RAF inhibitor PLX4032 inhibits ERK signaling and tumor cell proliferation in a V600E BRAF-selective manner. Proc Natl AcadSci U S A. 2010 Aug 17;107(33):14903-8.

Yang H., et al., RG7204 (PLX4032), a selective BRAFV600E inhibitor, displays potent antitumor activity in preclinical melanoma models. Cancer Res. 2010 Jul 1;70(13):5518-27.

McCubrey JA, Steelman LS, Chappell WH, et al. Roles of the Raf/MEK/ERK pathway in cell growth, malignant transformation and drug resistance.

Bergethon K, et al. ROS1 rearrangements define a unique molecular class of lung cancers (2012) J Clin Oncol 30: 863-70.

Lee SE, et al.Comprehensive analysis of RET and ROS1 rearrangement in lung adenocarcinoma. (2015) Mod Pathol 28: 468-79.

Rikova K, et al. Global survey of phosphotyrosine signaling identifies oncogenic kinases in lung cancer (2007) Cell 131: 1190-203.

Kutok JL, Aster JC. Molecular biology of anaplastic lymphoma kinase-positive anaplastic large-cell lymphoma. J Clin Oncol. 2002;20(17):3691-3702.

Iwahara T, et al. Molecular characterization of ALK, a receptor tyrosine kinase expressed specifically in the nervous system. Oncogene. 1997;14(4):439-449.

Soda M, et al. Identification of the transforming EML4–ALK fusion gene in non-smallcell lung cancer. Nature. 2007;448(7153):561-66.

Inamura K, et al. EML4–ALK fusion is linked to histological characteristics in a subset of lung cancers. J Thorac Oncol. 2008;3(1):13-17.

Choi YL, et al. Identification of novel isoforms of the EML4–ALK transforming gene in non-small cell lung cancer. Cancer Res. 2008;68(13):4971-76.

Koivunen JP, et al. EML4–ALK fusion gene and efficacy of an ALK kinase inhibitor in lung cancer. Clin Cancer Res. 2008;14(13):4275-83.

Shinmura K, et al. EML4–ALK fusion transcripts, but no NPM–, TPM3–, CLTC–, ATIC–, or TFG–ALK fusion transcripts, in non-small cell lung carcinomas. Lung Cancer. 2008;61(2):163-169.

Takeuchi K, et al. Multiplex reverse transcription–PCR screening for EML4–ALK fusion transcripts. Clin Cancer Res. 2008;14(20):6618-24.

Shaw AT, et al. Clinical features and outcome of patients with non-small-cell lung cancer who harbor EML4-ALK. J Clin Oncol. 2009;27(26):4247-4253.

Yi ES, et al. Correlation of IHC and FISH for ALK gene rearrangement in non-small cell lung carcinoma: IHC score algorithm for FISH. J Thorac Oncol. 2011;6(3):459-65.

McLeer-Florin A, et al. Dual IHC and FISH testing for ALK gene rearrangement in lung adenocarcinomas in a routine practice: a French study. J of Thorac Oncol. 2012;7(2):348- 54.

McGrath JP, Capon DJ, Smith DH, Chen EY, Seeburg PH, Goeddel DV, Levinson AD, 1983. Structure and organization of the human Ki-ras proto-oncogene and a related processed pseudogene. Nature 304 (5926): 501-6.

Lièvre A, Bachet JB, Le Corre D, et al.2006. KRAS mutation status is predictive of response to cetuximab therapy in colorectal cancer. Cancer Res. 66(8): 3992-5.

James RM, Arends MJ, Plowman SJ, et al.2003. KRAS Proto-Oncogene exhibits tumor suppressor activity as its absence promotes tumorigeneis in Murine Teratomas. Mol Cancer Res. 1: 820-5.

Douillard JY, Oliner KS, Siena S, et al.2013. Panitumumab-FOLFOX4 Treatment and RAS Mutations in Colorectal Cancer. The New England Journal of Medicine, 369 (1 1): 1023-34.

Roock WD, Jonker DJ, Nicolantonio FD, et al.2010. Association of KRAS p.G13D mutation with outcome in patients with chemotherapy-refractory metastatic colorectal cancer treated with cetuximab. JAMA, 304(16): 1812-1822011.
